# Determination of Three-Dimensional Corrective Force in Adolescent Idiopathic Scoliosis and Biomechanical Finite Element Analysis

**DOI:** 10.3389/fbioe.2020.00963

**Published:** 2020-08-13

**Authors:** Tianmin Guan, Yufang Zhang, Adeel Anwar, Yufen Zhang, Lina Wang

**Affiliations:** ^1^School of Mechanical Engineering, Dalian Jiaotong University, Dalian, China; ^2^School of Orthopedic Surgery, The Second Affiliated Hospital, Dalian Medical University, Dalian, China; ^3^Hebi People’s Hospital, Hebi, China; ^4^Xunxian Shantang Central Health Center, Hebi, China

**Keywords:** adolescent idiopathic scoliosis, brace, three dimensional correction, finite element modeling, corrective forces

## Abstract

**Aims:**

In this study we have considered the three dimensional corrective forces for correction of scoliosis by using a patient specific finite element model.

**Materials and Methods:**

An objective function of corrective forces in three-dimensional space was defined. Computed tomography images were used to reconstruct three dimensional model of scoliotic trunk. Computer aided engineering software Abaqus was used to establish finite element model of deformed spine and its biomechanical characteristics were analyzed. By adjusting magnitude and position of corrective forces, objective function was minimized to achieve best orthopedic effect. The proposed corrective conditions were divided into three groups: (1) thoracic deformity; (2) lumbar deformity; (3) both thoracic and lumbar deformities were considered.

**Results:**

In all three cases, the objective function was reduced by 58, 52, and 63%, respectively. The best correction forces point was located on convex side of maximum displacement of vertebral body.

**Conclusion:**

Using minimum objective function method, spinal deformity in three-dimensional space can be sufficiently reduced. This study provides scientific basis for design of a new corrective brace for treatment of scoliosis.

## Introduction

Adolescent idiopathic scoliosis (AIS) is a three-dimensional deformity of spine and ribs in adolescence, which results in trunk deformation. At present, brace is frequently used in treatment of AIS with Cobb angle 20° to 40°. This treatment modality is advocated as an effective method for treatment of scoliosis (Noonan and Weinstein, 1996; Goldberg et al., 2001; Weinstein et al., 2013). Traditionally designed models and fabrication methods of thoraco-lumbo-sacral orthosis (TLSO) are filled with plaster. Plaster is added or removed according to clinical experience. Finally, brace is thermoplastically formed on trimmed plaster cast. It’s shown that the correction effects of brace made by this method is often different from predicted effects and patients may have different degrees of flat back deformity in sagittal plane (Clin et al., 2010; Mauroy and Pourret, 2016). In order to quantify spinal deformity condition with and without braces, it is necessary to provide a set of effective parameters to analyze patient specific treatment effect of patients.

The finite element model (FEM) has been applied in biomechanical study of scoliosis treatment (Clin et al., 2011; Cobetto et al., 2016). In clinical series of 5 patients treated with Milwaukee braces, Andriacchi et al. (1976) has found that braces had better correction effects in patients with coronal deformity, but it increased incidence of flat back in sagittal plane. Goldberg et al. (2001) studied the treatment effects of Boston braces, and found that Boston braces not only improve coronal plane deformity but also minimizes rate of flat back syndrome. A recent study compared the correction effects of computer-aided design and manufactured braces with that of traditionally designed braces Cobetto et al. (2016). Moreover, braces obtained by computer aided design and manufacture technology are lighter and have better correction effects. Although many scholars have used FEM simulation to design personalized braces, but they mainly concentrated on improvements in coronal plane. 3D corrective forces of brace are not fully considered. Ghista et al. (1988) developed and presented a method for determining the stiffness of the patient’s spine prior to surgery. The effect of different instrumentation strategies was compared on the same patients with surgery simulator. Different strategies produced different results (Robitaille et al., 2009). Majdouline et al. (2009) built an objective function to define the optimal surgical instrumentation strategy using a computer model implemented in a spine surgery simulator. The research demonstrates the potential to optimize the planning of surgical instrumentation in AIS. Majdouline et al. (2012) defined correction objectives to get optimize instrumentation configurations. The ideal corrective effects of brace is lacking research.

In this study, deviation distance and rotation angle of scoliosis and normal spine in three directions (that is in coronal, sagittal and horizontal planes) are set as parameters to construct objective function. The biomechanics is simulated using FEM and analyzed the effects of brace correction. This method provides theoretical basis for designing a better correction brace.

## Materials and Methods

### Ethics

The patients/participants provided their written informed consent to participate in this study. For CT scan and identifiable images used for simulation study, the patients/participants informed and the ethical approval is not mandatory in our institution.

Patient specific 3D FEM was constructed from CT images data of AIS. The correction effects of different applied forces were analyzed by using biomechanical principles.

### Personalization of the Finite Element Model

The patient was an 11-year-old with AIS, having typical right thoracic and left lumbar scoliotic curvatures, with a mean thoracic Cobb angle of 36° and mean lumbar Cobb angle of 24°. The patient was scanned by helical CT scanner from T1 to pelvis. The maximum lateral bending displacement was in T9 and L3 vertebrae. In coronal plane, displacements were 56 and 38 mm whereas in sagittal plane these were 46 and 35 mm respectively. The maximum rotation angle of ribs was 13° and kyphotic displacement of ribs was 23 mm. A 3D model of AIS was established by using medical image processing software (Mimics 19.0; Materialise, Leuven) from DICOM CT images. Spine model was extracted according to bone threshold and then region growing tool was used to segment the different regions. Finally, 3D geometric model of vertebral bodies, ribs, and pelvis was obtained for simulation. Threshold of the irregular shaped intervertebral disks was close to muscles, so it was difficult to extract directly from CT images. Upper and lower surfaces of vertebral body were extracted and corresponding cylinders were established. 3D model of intervertebral disks was obtained using Boolean operation in 3-Matics. 3D spine model was optimized by using reverse engineering software (Geomagic studio 2013; Geomagic, America) to facilitate the next biomechanical simulation. In Geomagic studio we denoised and smoothened to reconstruct a 3D model. The software (Hypermesh13.0; Altair; America) was used to mesh 3D model.

**FIGURE 1:**
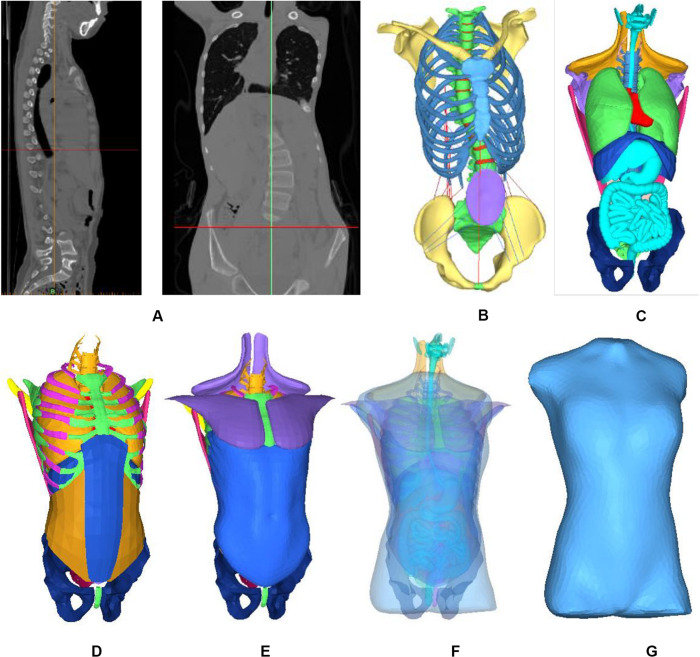
Finite element model of patient trunk. **(A)** Constructing the internal structure of human body by using three-dimensional reconstruction technology of CT data. **(B)** Skeleton model, **(C)** Organ model, **(D)** Soft tissue, **(E)** Muscles, **(F)** All model, and **(G)** Finite element model.

The patient’s trunk FEM is shown in Figure 1. According to CT date, bones, organs and soft tissues models were built. Threshold segmentation algorithm and region growing in Mimics were used for data extraction. Thresholding tool was used to extract the skin tissue, and region growing and edit masks tools were used to segmentation. The fuzzy boundary between the muscles in CT makes it difficult to extract the muscle geometry model. The geometric model of the main abdominal muscles constructed extracted from CT slices. The missing pieces established in the Geomagic software based on *systematic anatomy* (Liu, 2005) and *ultrasound imaging of the musculoskeletal system* (Wang et al., 2007). The majority of human muscles are skeletal muscles, which generally span one or more joints, and their ends are attached to two bone surfaces. The skeletal muscles start at one point and end at the other point. Skeletal muscles move by pulling bones. When the muscle is stretched, the abdomen stretches narrower; when the muscle contracts, it shortens and becomes thicker (Zatsiorsky and Prilutsky, 2012). According to the principle of anatomy (Elaine, 2015), the starting and ending points of skeletal muscles were defined. Skeletal muscles built in Geomagic software. The cross-sectional area of each ligament was obtained from Ref (Goel et al., 1995). Bones included sternum, ribs, costal cartilage, spine, intervertebral disk, and pelvis. The density of bone was simulated by an elastic shell element. The cancellous bone was simulated by a hexahedral elastic element. Spine combines vertebral, nucleus pulposus and endplate. Vertebral and nucleus pulposus were built in hexahedral elastic unit. Endplate was built in elastic shell element. Pelvis combines ilium, pubis, ischia, sacrum and cartilage. All be defined elastic hexahedral element. Heart, liver, spleen, lung, and kidney were defined as viscoelastic hexahedral units. The stomach, large intestine and bladder were defined as a viscoelastic hexahedral unit. They were internally defined as two layers of viscoelastic hexahedral elements. Abdominal muscles include external oblique, internal oblique, rectus abdominis, transverse abdominis, psoas major, erector spinalis and latissimus dorsi. Muscle was defined as a viscoelastic hexahedral unit. Ligaments were fibrous elastic connective tissue, simulated by elastic shell elements. Mechanical properties of anatomical structures were taken from published data (Table 1) (Roberts and Chen, 1970; Brown et al., 1981; Sharma et al., 1995; Wang, 1995; Aubin et al., 1996; Howard et al., 1998; Stephen, 2003; Périé et al., 2004; Clin et al., 2007, 2011). Two models was face to face contact. 3D model was processed and simulated in CAE (computer assisted engineering) software (Abaqus 6.14-2; Dassault; France).

**TABLE 1:** Material properties of each part.

Components	Young’s modulus *E* 10^6^ Pa	Poisson’s ratio *ν*		Density *ρ* kg/m^3^		Material model	References
Cortical bone	12000	0.3		1830		Elastic-plastic		
Cancellous bone	100	0.2		1000		Elastic-plastic	Brown et al., 1981
Posterior structure	3500	0.25		1000		Elastic-plastic	
Nucleus	1	0.49		1040		Elastic	
Annulus	4.2	0.45		1000		Elastic	Sharma et al., 1995
Endplate	24	0.4		1200		Elastic	
Rib	5000	0.1		1100		Elastic	Roberts and Chen, 1970
Pelvis	5000	0.2		1000		Elastic	
Sacrum	5000	0.2		1200		Elastic	Clin et al., 2007, 2011
Costal cartilage	480	0.1		1600		Elastic	Périé et al., 2004
Ligament	31.5	0.45		1000		Elastic	Yamamoto et al., 1989
Skin	31.5	0.42		1000		Elastic	
Muscle	1.33	0.14		1000		Elastic	Wang, 1995

**Components**	**Density *ρ* kg/m^3^**	**K/10^6^ Pa**	**G_0_/10^6^ Pa**		**G_∞_/10^6^ Pa**	**Material model**	**References**

Heart	1000	2.6	440		150	Viscoclastic
Liver	1100	2.8	230		44	Viscoclastic	
Spleen	1100	2.8	230		44	Viscoclastic	Yamamoto et al., 1989
Kidney	1100	2.8	230		44	Viscoclastic	
Lung	600	2.2	0.02		0.075	Viscoclastic	
Sternum	2000	9592	4423		2.3	Elastic	Stephen, 2003

### Objective Function

#### Normal Spine Model

Due to symmetry of human body, there was no hump in coronal plane of the normal spine and ribs were symmetrically in horizontal plane of midline of spine (Stokes, 1994). In order to obtain curve of normal spine in sagittal plane, 12 healthy adolescents with no medical history of spinal disorders were selected, with an average age of 12.0 years consisting of six males and six females. The results in form of normal curve of spine were obtained.

#### Defining Parameters

**FIGURE 2:**
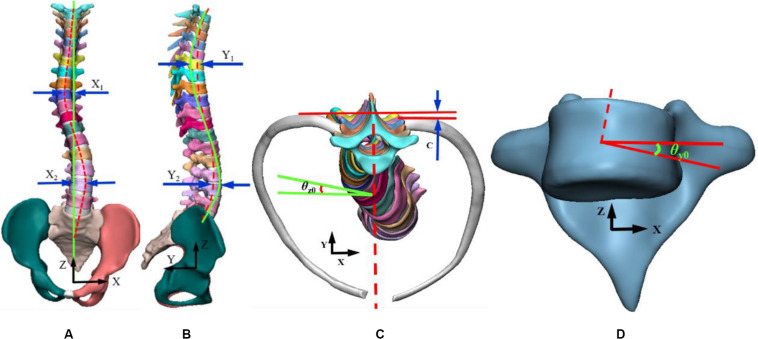
Representation of variables in a three-dimensional view. **(A)** Coronal plan, **(B)** Sagittal plan, **(C)** Transverse plan, **(D)** Vertebral rotation angle.

Combined with the research of scholars (Wynarsky, 1988, Wynarsky and Schultz, 1991; Gignac et al., 2000; Majdouline et al., 2012) and spinal deformities orthopedic parameters in 3D plane, the objective function was optimized. In coordinate system, coronal axis was *X* axis, sagittal axis *Y* axis and vertical axis as *Z* axis. Curvature curve was defined by connecting the center of each vertebral body in *Y* axis. Curvature was defined based on the orientation of vertebral endplates in *X* axis. Similarly, normal spinal curve was defined. In this work, objective function was consisted of seven variables; displacement distance between scoliotic and normal spinal curves in thoracic and lumbar regions in coronal plane as X_1_, X_2_, and in sagittal plane as Y_1_, Y_2_ respectively. The objective function was non-dimensionalized to unify the units of each term. The reference was defined as the height of L5. The height is 21 mm, marked as a^∗^. Distance between left and right ribs kyphosis in horizontal plane was defined as C. The rotation angle of first scoliosis rib was θz_0_, and initial rotation angle of vertebral body was θy_0_. These variables are represented in the 3D view interface as shown in Figure 2.

Since geometric variables can be positive or negative, the square of displacement was chosen to be square and square root to ensure that the numerical value is positive. The objective function is expressed as follows:

$$W=\alpha \frac{\sqrt{\left(X_{1}^{2}+X_{2}^{2}\right)}}{\sqrt{a^{*}){}^{2}}}+\beta \frac{\sqrt{\left(Y_{1}^{2}+Y_{2}^{2}\right)}}{\sqrt{{\left(a^{*}\right)}^{2}}}+\gamma \frac{\sqrt{C^{2}}}{\sqrt{{\left(a^{*}\right)}^{2}}}$$

(1) $$+\delta \frac{\sum_{i=1}^{N}\sqrt{{\left(\theta_{z0}+d\theta_{z}\right)}_{i}^{2}}}{\sum_{i=1}^{N}\sqrt{{\left(\theta_{z0}\right)}_{i}^{2}}}+\varepsilon \frac{\sum_{i=1}^{N}\sqrt{{\left(\theta_{y0}+d\theta_{y}\right)}_{i}^{2}}}{\sum_{i=1}^{N}\sqrt{{\left(\theta_{y0}\right)}_{i}^{2}}}$$

(1-a) $$\alpha =\frac{\sqrt{\left(X_{1}^{2}+X_{2}^{2}\right)}}{\sqrt{\left(X_{1}^{2}+X_{2}^{2}\right)}+\sqrt{\left(Y_{1}^{2}+Y_{2}^{2}\right)}+\sqrt{C^{2}}}$$

(1-b) $$\beta =\frac{\sqrt{\left(Y_{1}^{2}+Y_{2}^{2}\right)}}{\sqrt{\left(X_{1}^{2}+X_{2}^{2}\right)}+\sqrt{\left(Y_{1}^{2}+Y_{2}^{2}\right)}+\sqrt{C^{2}}}$$

(1-c) $$\gamma =\frac{\sqrt{C^{2}}}{\sqrt{\left(X_{1}^{2}+X_{2}^{2}\right)}+\sqrt{\left(Y_{1}^{2}+Y_{2}^{2}\right)}+\sqrt{C^{2}}}$$

(1-d) $$\delta =\frac{{\left(\theta_{z0}\right)}_{i}}{10}$$

(1-e) $$\varepsilon =\frac{{\left(\theta_{y0}\right)}_{i}}{10}$$

Where α, β, γ and δ, ε are the weightings of distance and rotation in coronal, sagittal and horizontal planes respectively.

The interval ranges from 0 to 1, N is total number of vertebrae, and *d*θ is increment of relative initial rotation value of each vertebra. In order to fully consider the lateral bending of vertebrae and ribs, rotation angles of each rib and vertebrae were counted. The optimal solution of objective function was to reach the minimum value that is 0 in equation.

The optimal scoliosis correction curve was considered when body was in upright position whereas lateral displacement, tilt angle and axial rotation of spine in 3D space were all 0. In actual clinical scoliosis correction process, due to complexity of muscles, skeleton, and other structures, the correction often cannot achieve ideal state. Therefore, in treatment of scoliosis, goal is to minimize the objective function. It can be done to minimize each variable in space as much as possible. In patients with bilateral bends, displacement of coronal plane was statistically larger than that of sagittal plane (Cobetto et al., 2016). Clinically, physicians usually consider coronal deviation instead of vertebral rotation, ribs rotation angle. When the patient’s Cobb angle is greater than 10°, brace treatment is required. So the δ and ε are defined as the 10° standard. Set two groups of weights: a; δ is 0.5, ε is 1 and b; δ is 0.8, ε is 0.5. In order to distribute the weight of each variable reasonably, which is to define the proportion of each deformation amount to the total deformation amount. The weights were distributed to make the proportion of each deformation amount was variable. The migration distance larger, the weight bigger.

### Loading Condition

**FIGURE 3:**
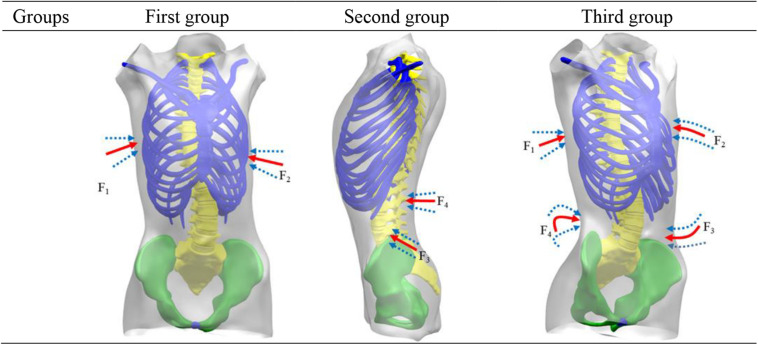
Force position.

According to the Hueter–Volkmann law (Castro, 2003), pressure on the epiphysis of spine inhibits the growth of epiphysis. A three-point system is formed by a force and two counterforces applied proximally and distally to the first one. The direction of the forces and counterforces are always from lateral to medial, but the pads (mainly lumbar and thoracic) providing the vector forces are oriented in an oblique plane rather than in a single frontal plane, so they will also provide the forces for derotation in the transversal plane (Rigo and Jelacic, 2017). Three point force principle is applied to scoliosis model to achieve correction effects in 3D space. Applied force was divided into three groups: (a) F_1_ and F_2_ were applied to thoracic vertebrae and ribs to examine correction of thoracic vertebrae in coronal axis and ribs kyphosis. (b) Application of F_3_ and F_4_ in position of lumbar scoliosis, measuring lumbar vertebral correction; (c) Four sets of correction forces, F_1_, F_2_, F_3_, and F_4_, were applied to position of lateral bend at same time. In coronal plane, F_1_ and F_3_ correction forces were applied to thoracic and lumbar vertebrae in coronal plane. In horizontal plane, deformity of vertebral rotation was improved by applying F_2_ correction forces on kyphotic ribs. In sagittal plane, F4 correction forces were applied to posterior lumbar spine to alleviate flat back and to maintain normal physiological curvature. The specific application of force is shown in Figure 3. Proposed forces were transmitted to spine through thoracic and lumbar soft tissues. In the case of thoracic scoliosis, the brace was located on the ribs connected with the apex vertebra. The forces traveled through ribs to spine. For lumbar scoliosis, brace applied to the spine through paravertebral muscles.

Literature concerning non-surgical treatment of scoliosis showed that initial maximum displacement of thoracic and lumbar vertebrae was within 100 mm and rotation angle of ribs is within 40° (Wynarsky and Schultz, 1991). Greater displacement can combine more patients. In clinical, doctors can make a preliminary examination based on the patient’s data. In order to make this study more practical, the maximum displacement of vertebrae and maximum rotation angle of ribs were set at 200 mm and 50° respectively.

### Finite Element Analysis

When body is under high pressure, it will not only produce discomfort but also cause pressure ulcers. In order to ensure that local pressure was within tolerance range, it was necessary to select a reasonable stress area. Visser et al. (2012) used visual analog scales and pressure sensors to measure pressure generated by braces. Other researchers measured pressure on trunk of in-braces patients in range of 10 cm^2^ and withstanding 10000 pa pressure while contact point force was kept within 10 N (Pham et al., 2008; Raux et al., 2014; Hoogendoorn et al., 2017). Cobetto et al. (2016) collected the pressure between brace and patient’s body surface and gave a withstanding threshold of 35000 pa. The influence of human active and passive forces on modification was considered comprehensively, and correction forces were guaranteed in range of human pain threshold. The applied range of force was 0 ∼ 100 N, and the applied area was 64 ∼ 225 cm^2^ (Desbiens-Blais et al., 2012; Cobetto et al., 2016).

### Convergence Conditions

Internal and external geometries were linked together. Using an iterative non-linear resolution method, applied forces on selected vertebrae lead to correction of external trunk model (Zhang and Mak, 1999; Ferziger et al., 2002). The simulation boundary conditions included a fixed pelvis in rotation-translation. T1 was limited to transverse plan movements. According to scoliosis curve of patient, the values of variables in objective function were calculated, and initial variables were taken as original values. By adjusting magnitude of correction forces, the deformation of objective function was obtained. As the initial value of next iteration, results of the previous step were iterated until convergence conditions were satisfied. Convergence conditions used were as: (1) total number of iterations exceeding 200 steps; (2) variation of variables in two iterations was within 2 mm; (3) difference between two iterations was less than 1 N, accounting for about 1% of the force range. When convergence condition was reached, the iteration was terminated.

### Grid Independence Verification and Finite Element Model Verification

Ferziger et al. (2002) pointed out that with the improvement of grid quality, the error of performance prediction will gradually decrease. The number of grids has a great influence on results of numerical calculation. Only when number of grids increases has little influence on calculation results, the numerical results will be meaningful. The indicators were stress and displacement at T8 in trunk model. Four groups of different grids are divided and simulated respectively to get corresponding stress and displacement.

Table 2 shows when number of grids is C and D, there is little difference in transformation of simulation results. Considering computational efficiency and simulation error, it can be approximated that the number of grid C can meet requirement of irrelevance of number of grids, and it can be used as computational grid in this study. Patient model was divided into 394273 elements and 323021 nodes.

**TABLE 2:** Grid number.

Project	Grid number			
	A (32588)	B (162293)	C (263673)	D (500506)
Stress pa	3668	9377	1085	1088
Displacement mm	3.291	1.257	1.290	1.294

**FIGURE 4:**
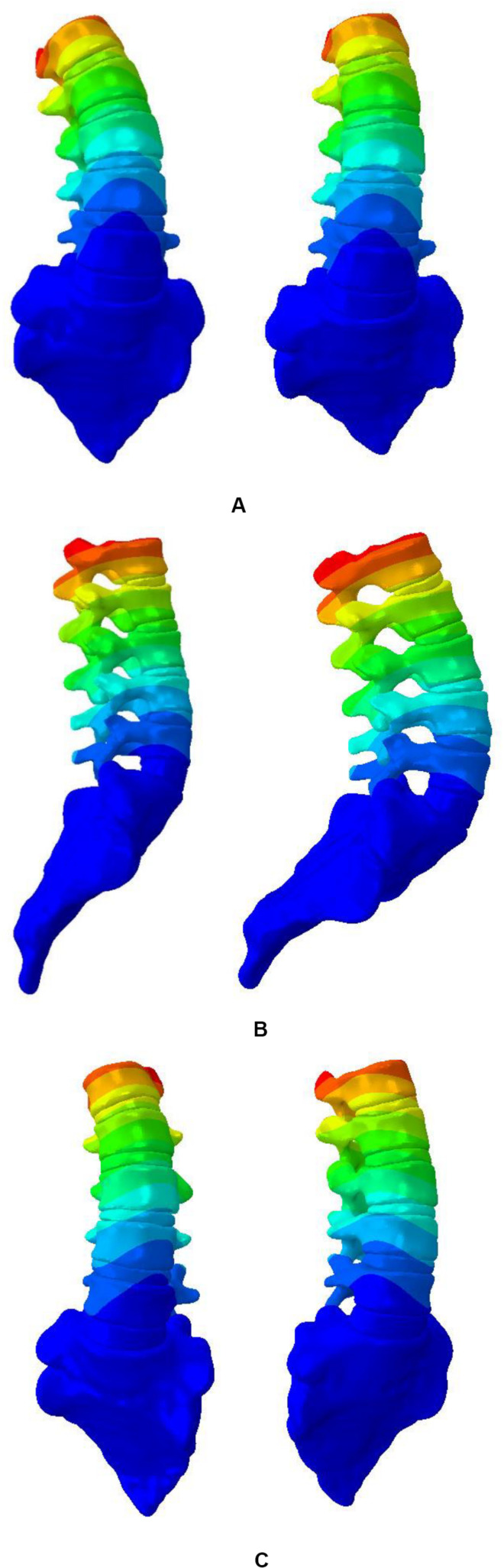
Displacement nephogram. **(A)** Left and right side bending, **(B)** Flexion and extension, and **(C)** Twist around.

**FIGURE 5:**
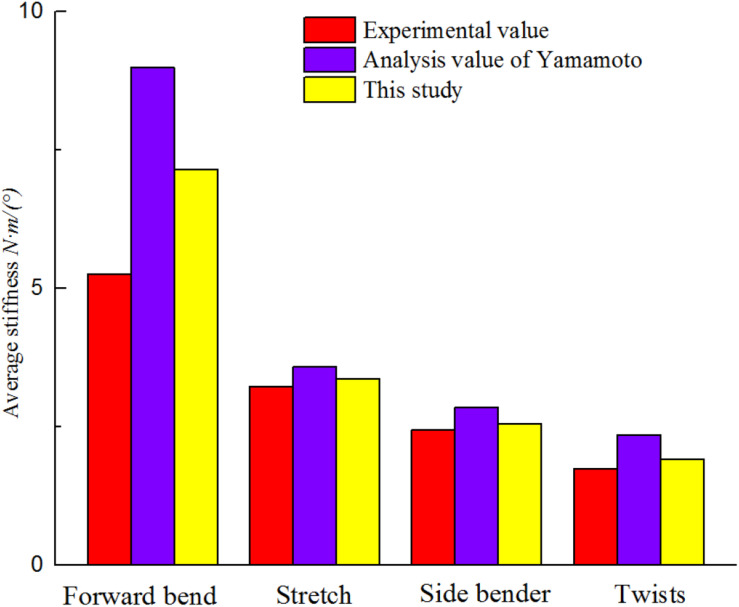
Finite element model verification.

The biomechanical characteristics of the FEM need to be consistent with the actual situation of the patient. The established model needs to be validated before performing simulated biomechanical analysis. Validity of the established FEM of scoliosis was verified. The displacement of L1–L5 vertebral was simulated under 10N • m torque in L1 top (Figure 4). At present, there is no experimental data on biomechanics of AIS’s whole spine (Nie et al., 2009). L1–L5 segments were used to simulate changes of vertical compression, forward bending, backward extension and lateral bending under 10N • m external moment of force. It is in the middle of average comparison results, as shown in Figure 5 (Yamamoto et al., 1989; Heuer et al., 2007).

**FIGURE 6:**
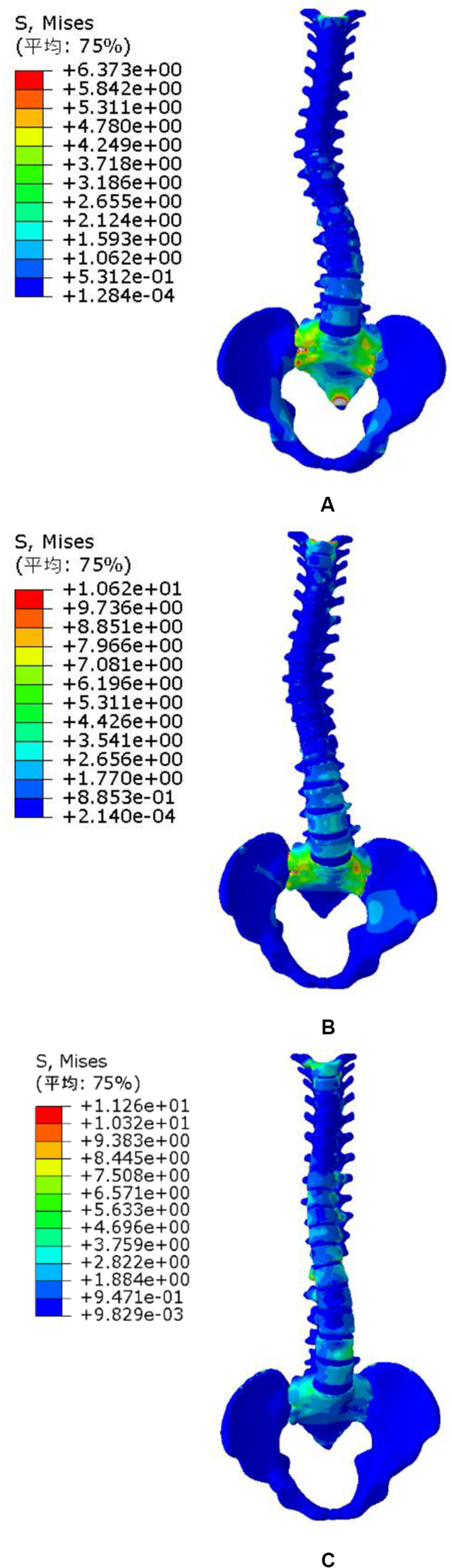
Spine stress under different strap tension. **(A)** 20N trap tension, **(B)** 40N trap tension, and **(C)** 60N trap tension.

The pressures generated from the patients on the torso to spine were also evaluated and compared to the simulations showed in Clin et al. (2011). The same orthopedic force was applied to the established trunk model. The changes of spinal stress are shown in Figure 6. Compared with stresses in the 20N, 40N, and 60N strap tension (Figure 6). According to the torso forces applied on the trunk by Mac-Thiong et al. (2004), the stress of this model was simulated. When the torso is subjected to strap tension transmit to the scoliosis spine, the stress cloud of spine is compared with Nei (2009) and Clin et al. (2011). Same forces were loaded in FEM. Stress nephogram of T1-S1 under different strap tension shown in Figure 6. Figure 9 shows the spine in the frontal and sagittal planes before and after the simulations with strap tensions of 20N, 40N, and 60N. We simulated in our model in each strap tension, the stresses generated on the spine are in the middle of the values of Clin and Nie. With the increase of strap tension, the spine in the coronal and sagittal plane of orthopedic effect is obvious. The Cobb angle decreases with the increase of the tightening force. Therefore, the scoliosis model established in this study is reliable and reasonable, and can be used for finite element analysis.

## Results

The degrees of coronal correction of thoracic and lumbar vertebrae were 58 and 52% respectively. Taking two groups of weightings as examples, the changes of each index under different weights were compared. The change was related to weight setting, which showed that weight setting was reasonable. Table 3 shows that deformities of patients in 3D space have been corrected to varying degrees compared with initial value. Finite element simulation ignores some external conditions, which is more corrective than clinical trials. After applying correction forces on sagittal plane to alleviate flat back phenomenon, it was improved which is close to normal physiological spinal curve. The best correction forces were: F1 of 58N, located at seventh rib on right side of model; F2 of 36N, located in front of left fifth rib; F3 of 41N on left side of third lumbar vertebrae and F4 of 23N on posterior part of second lumbar spine.

**TABLE 3:** Index parameter.

Parameter (mm)	Initial value	First group		Second group		Third group	
		δ0.5 ε1	δ0.8 ε0.5	δ0.5 ε1	δ0.8 ε0.5	δ0.5 ε1	δ0.8 ε0.5
**Cobb (°)**							
Thoracic	36	25	23	32	30	14	15
Lumbar	24	26	27	16	17	10	12
**Sagittal plane (°)**							
Thoracic	34	33	35	36	35	36	35
Lumbar	35	36	37	34	35	38	37
Rib kyphosis	31	35	31	34	33	45	43
Lumbar lordosis	38	32	33	36	37	31	32
X_1_	63	32	30	36	34	15	12
X_2_	−32	−25	−26	−16	−19	−10	−13
Y_1_	−34	−18	−16	−22	−24	−8	−7
Y_2_	26	16	17	9	8	4	6
C	23	12	11	20	19	6	7
θ_z0_(°)	13	6	5	11	10	5	5
θ_y0_(°)	14	7	8	10	11	4	6

The simulation results showed that maximum displacement of first group was at T8 (the maximally deformed vertebral body) and the displacement was 74.1 mm. Through F2, thoracic scoliosis ranges from 56 to 16 mm. In second group, F3 and F4 correction forces were applied to L3 and posterior lumbar spine respectively. Lumbar scoliosis ranges from 38 to 11 mm. Because in second group only lumbar correction forces were applied, the side bending of chest was affected and improvement effect was poor. In third group, when thoracolumbar and lumbar correction forces were applied simultaneously, overall scoliosis was corrected and spinal curve was close to that of normal spine curve. Scoliosis was improved in 3D space and increased in vertical direction to a certain extent. When correction force was 20N, stresses on convex side and concave side of disk between T7 and T8 were 120000 and 93000 Pa respectively. When correction force was increased to 40N, stress on convex side reached to 153000 Pa and stress on concave side was 84000 Pa. Stress on convex side and back side of vertebral body was larger than that was on concave side, and stress level of cortical bone was higher than that of cancellous bone. The maximum stress value appeared in cortical bones of thoracic and lumbar segments. Disks with greatest stress were located on the most convex side of deformed spine, and maximum stress value was between T7 and T8. It showed that objective function has significance in correction of scoliosis in 3D space.

**FIGURE 7:**
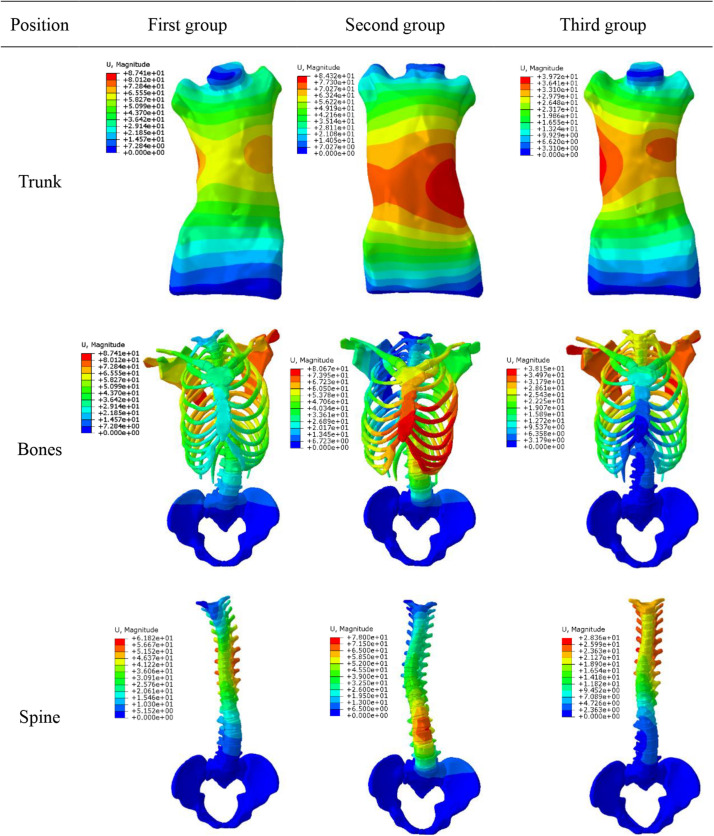
Displacement variables.

**FIGURE 8:**
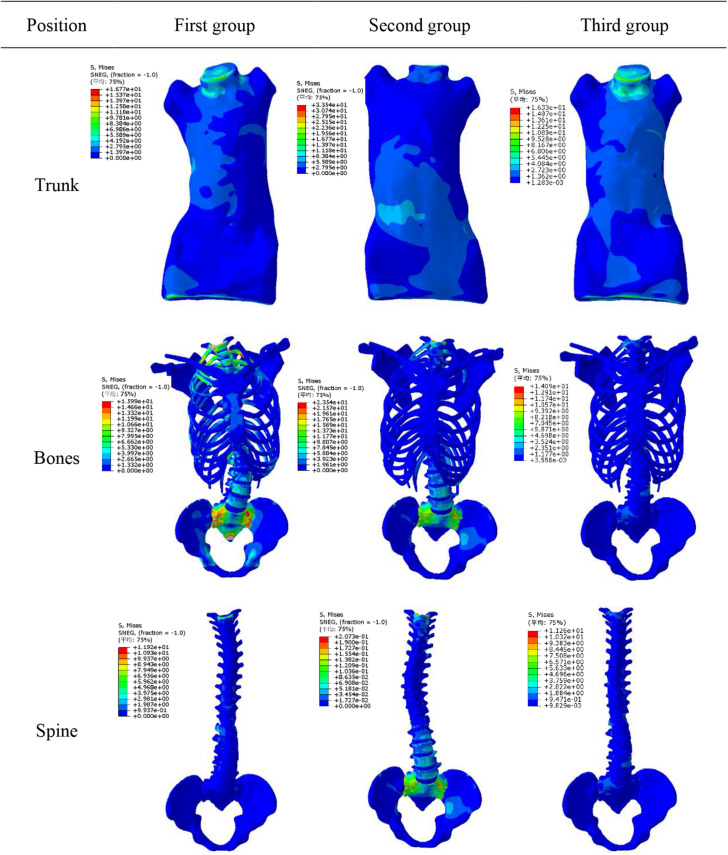
Stress variation.

Figure 7 shows displacement variables with different forces. First group maximum displacement was in the apex of the thoracic vertebra. Second group maximum displacement was in the apex of the lumbar vertebra. The thoracic and lumbar vertebrae were applied to the third group, respectively. Through the transmission of orthopedic forces, the scoliosis was corrected. The whole spine is close to the normal spine. Stress as well as displacement changes. Due to constraints on upper and lower boundaries of model, the maximum stress was at upper and lower ends of trunk (Figure 8).

## Discussion

In this study, a new objective function is proposed, which considers correction of spine and ribs in 3D space more comprehensively using the braces. This provides a theoretical basis to design much better braces with higher efficiency. The defined aim index was as: (a) Cobb angle of thoracic and lumbar lateral bending in coronal plane; (b) sagittal rib kyphosis and lumbar lordosis; (c) values of variables in objective function were the lateral bending distances on each plane. By optimizing iteration calculation, optimal correction forces can be obtained after convergence condition is reached. According to our findings, application of different correction forces in 3D space can reduce rate of flat back phenomenon.

This study shows that correction results in coronal plane are similar to those achieved by clinical wearing braces. However, it can reduce adverse effects of traditional braces in sagittal plane and horizontal plane, such as flat back in sagittal plane and rotation in horizontal plane. Although this simulation is a correction effect produced at moment of application of force, it has a certain correlation with long-term treatment. Chase et al. (1986) advocated that there was a correlation between instantaneous correction and long-term treatment. When defining the objective function, seven deformations were defined to consider displacements and rotation in 3D space. The weightings definition of a function was related to value of variable, and weight changed when variable changed. In order to avoid large errors in simulation process, convergence conditions were set up in this study. However, residual deformities or overcorrection were allowed in course of correction. In actual clinical correction process, actual orthotic forces were not consistent with the simulated force. In the simulation process, the model is simplified to a certain extent relative to the actual model of the human body. Generally, simulation results are better than clinical corrections. However, simulation can predict the actual orthopedic situation and has a guiding role in clinical treatment. The maximum stress value in three groups was 354000 pa, which is lower than maximum stress threshold that human body can bear (Cobetto et al., 2014). Practically, human body can be adjusted automatically because there is no constraint of upper and lower boundaries, and stress is smaller than simulation results. In order to make the simulated value closer to the actual value, the convergence range needs to be smaller. In treatment of scoliosis patients, physicians can measure 7 variables according to actual situation of patients. According to individual condition of patients, weight of objective function is individually set to get the best correction effects.

In simulation of spine model, as 3D correction forces increased, Cobb angle of thoracolumbar segment gradually decreased and rotation angle of vertebral body decreased too. Combined correction effects of combined forces were better. After applying correction forces, stress at intervertebral disks in deformed area changed significantly.

**FIGURE 9:**
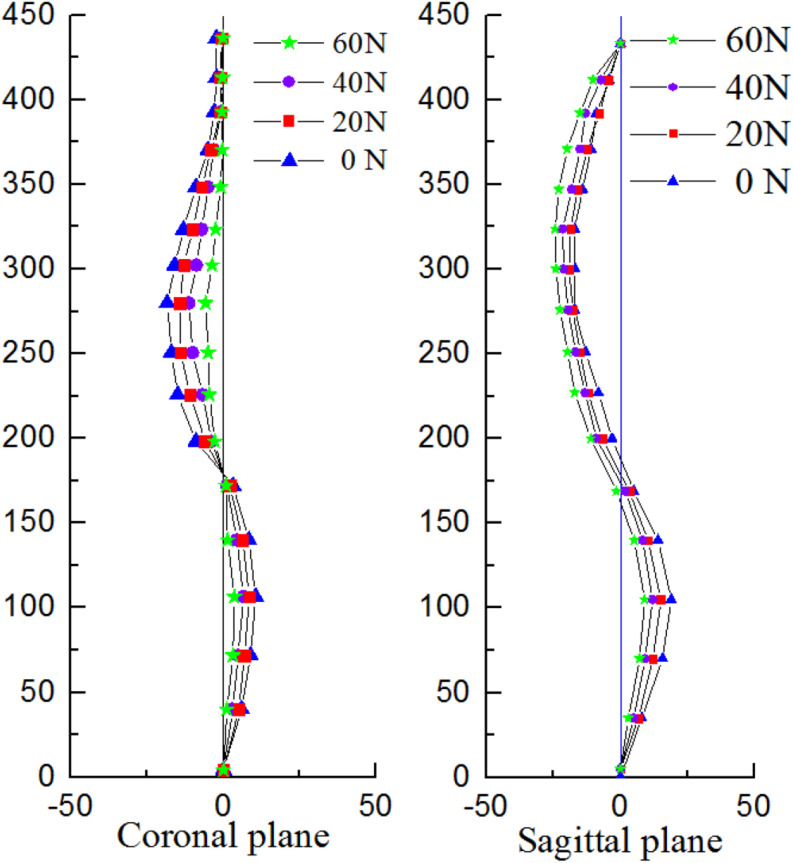
Changes in the curvature of the spine with different correction forces.

According to Hueter-Volkmann law (Castro, 2003), when the epiphysis of the spine is under pressure, the growth of the epiphysis will be inhibited, otherwise the epiphysis will accelerate the growth. 3D correction forces compensated the deficiency in 2D plane correction, and did not produce side effects such as flat back. The overall correction effects were better than that was reported in previous work (Gignac et al., 2000). Changes in coronal and sagittal spine curves are shown in Figure 9.

Based on geometry of spine and ribs in AIS patients, an optimal method of 3D correction forces are proposed in this work. In long run, this method can be used to design and optimize position of braces, pads and so on to achieve correction aim in 3D space. This method can effectively improve efficiency and therapeutic effects in designing and optimizing 3D corrections. It can avoid surgical treatment and reduce burden on patients. It has good clinical significance and prospects. The limitations on this study are fewer patient and some factors not yet been explored (i.e., muscular activation, spine stiffness, deformity magnitude, and differences of patients, etc.). The next step is to apply the objective function to more patients to analyze the reliability of the function.

## Data Availability Statement

Patient specific 3D finite element model was constructed from CT images data of AIS. The correction effects of different applied forces were analyzed by using biomechanical principles.

## Ethics Statement

The patients/participants provided their written informed consent to participate in this study. For CT scan and identifiable images used for simulation study, the patients/participants informed and the ethical approval is not mandatory in our institution.

## Author Contributions

TG, YFaZ, and AA drafted the manuscript and conceived of the study. YFeZ and LW were also responsible for statistical analysis. All the authors contributed to the article and approved the submitted version.

## Conflict of Interest

The authors declare that the research was conducted in the absence of any commercial or financial relationships that could be construed as a potential conflict of interest.
